# Why? What? When? Utility of 4DCT in the preoperative imaging algorithm of primary hyperparathyroidism

**DOI:** 10.4102/sajr.v29i1.3094

**Published:** 2025-06-30

**Authors:** Swati P. Joshi, Jai Chowdhary, Sanyukta Gupta, Vineet Mishra

**Affiliations:** 1 Department of Radiodiagnosis, Mahatma Gandhi Medical College and Hospital, Jaipur, India

**Keywords:** 4DCT, adenoma, parathyroid, primary hyperparathyroidism, multiglandular

## Abstract

**Background:**

4-Dimensional computed tomography (4DCT) is a specialised examination used to locate diseased parathyroid glands in a confirmed case of primary hyperparathyroidism.

**Objectives:**

To define the role of 4DCT as a first-hand diagnostic tool in locating the abnormal parathyroid gland.

**Method:**

A retrospective cohort study of 44 patients with primary hyperparathyroidism was performed. Patients with preoperative 4DCT findings and surgical findings with histopathological results were included in the study to assess the effectiveness of 4DCT in locating the diseased parathyroid glands.

**Results:**

Of the 44 patients who underwent 4DCT, operative findings of three patients were discordant with the 4DCT findings. The calculated sensitivity of 4DCT was 93%. 4DCT was able to identify lesions in ectopic locations in two cases and missed one case in an intra-thyroidal location, misinterpreted as a suspicious thyroid lesion. The sensitivity of 4DCT in detecting multiglandular disease was 75%. Of the diagnosed parathyroid lesions, 52.1% were located on the left, 35.4% on the right and 12.5% were located bilaterally. Additionally, 76% were seen inferiorly and 24% were seen superiorly.

**Conclusion:**

4DCT has high utility in the presurgical localisation of the eutopically or ectopically placed diseased parathyroid glands in single and multiglandular disease and also provides additional anatomical details.

**Contribution:**

4DCT identified additional findings such as aberrant origin of right subclavian artery, which is an important pre-operative finding for the surgeon to be aware of. This study contributes to the existing literature on the role of 4DCT.

## Introduction

**FIGURE 1:**
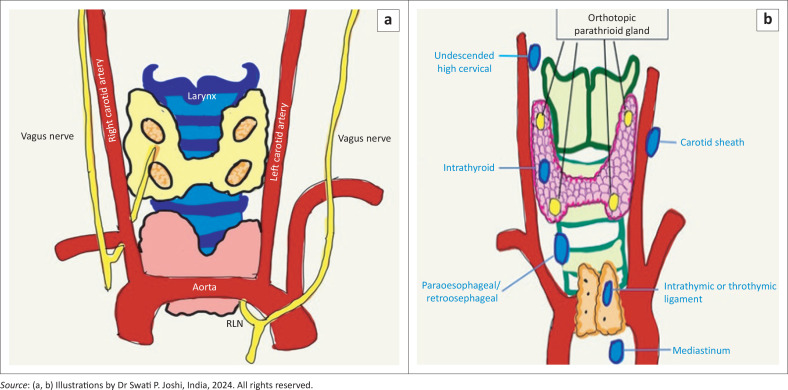
Schematic depiction of the anatomy of the (a) eutopic parathyroid glands and the recurrent laryngeal nerve (RLN) and (b) the ectopic parathyroid glands.

The parathyroid glands are tiny, oval-shaped glands, located next to the thyroid gland. The anteroposterior diameter of these glands is 1 mm – 2 mm, length 6 mm and transverse diameter 3 mm – 4 mm.^1^ Typically, these glands are located in close proximity to the thyroid gland, with two located superiorly and two inferiorly. Imaging does not always demonstrate these glands; therefore, if a parathyroid gland is identified, it suggests a pathological gland (Figure 1b).^2^

The superior parathyroid glands are usually seen along the posterior aspect of the thyroid gland, near the junction of the cricoid and thyroid cartilages. They may be located in ectopic sites such as in an intra-thyroidal location or in proximity to the carotid vessels and sheath. If these enlarged glands extend to the retro-oesophageal or retro-pharyngeal locations posteriorly and inferiorly, they are commonly referred to as acquired ectopia.^3^ The inferior parathyroid glands are seen along the lateral, posterior or lower aspect of the lower pole of the thyroid gland or in ectopic locations, extending from the angle of mandible to the anterior mediastinum, in proximity to the carotid sheath or within the thyroid gland itself.^3^ The recurrent laryngeal nerve (RLN) plane is seen to lie along the tracheo-oesophageal groove with the superior glands lying along its posterior aspect and the inferior glands lying along its anterior aspect (Figure 1a).^4,5^

Primary hyperparathyroidism leads to hypercalcaemia with disturbances in calcium and phosphate meta-bolism. The diagnosis of primary hyperparathyroidism is determined on the basis of raised calcium levels above normal (> 10.2mg/dL) and non-suppressed serum parathyroid hormone (PTH) levels (> 30pg/mL). It is one of the most commonly encountered endocrinological disorders after diabetes and thyroid disease. Various studies in the Indian population have reported primary hyperparathyroidism in 2.5 out of 1000 individuals.^6,7,8^ Females seem to be more frequently affected, with a male to female ratio of 1:2.1 in the population,^9^ and a higher incidence especially in the post-menopausal group above 50 years of age. Single adenomas are the leading cause in primary hyperparathyroidism, present in 85% of instances.^8^ Other less frequently seen causes are multiglandular disease from multiple adenomas (4%), parathyroid hyperplasia (6%) and, less commonly, parathyroid carcinoma (1%).^10,11^

The criteria for single-gland disease are when histopathologically, there is a single adenoma or parathyroid hyperplasia, and postoperative normalisation of the previously raised calcium levels is noted. Similarly, the criteria for multigland disease are when histopathologically, there are more than one parathyroid adenomas or there is hyperplasia or hypercalcaemia persists after surgical removal of a single diseased gland.

The role of the radiologist is to anatomically map the parathyroid lesions, facilitating the planning of parathyroid surgery for patients suffering from hyperparathyroidism. In situations where neither ultrasonography (USG) nor sestamibi scanning is positive, 4-dimensional computed tomography (4DCT) can indicate the presence of the diseased gland or indicate that several glands are abnormal.

All symptomatic individuals with primary hyperparathyroidism are advised to undergo surgery. Patients who have no symptoms but have increased calcium levels, approximately 1 mg/dL above normal, are also advised to undergo surgery according to the American Association of Endocrine Surgeons.^2^

Minimally invasive surgery is where pre-operatively, the adenoma is removed through a small incision. If the expected drop in PTH levels occurs after 10 min post-removal of the single adenoma, the rest of the glands are left untouched and the surgery is considered to be complete. If the PTH levels do not decrease by more than 50% within 10 min of excising the parathyroid lesion, it indicates the possibility of multiglandular disease or persistent adenoma.^2,12^ In our centre, we also perform a frozen section biopsy in addition to intraoperative PTH levels, which help us determine whether the surgery is complete or further parathyroid gland exploration is necessary.

Minimally invasive and targeted surgery has several advantages, such as reduced surgery duration and surgery-related morbidity, better cosmesis, shorter hospital stays and decreased expenses.^13^ Cure rates are similar with both minimally invasive surgery and bilateral neck exploration.^14^ The complications that can be seen in bilateral neck exploration are injury to the RLN and hypoparathyroidism.^15^

This study assessed the role of 4DCT imaging in delineating the involvement of single or multiglandular disease, highlight relevant and important anatomical details and describe any associated or coincidental findings affecting the surgical decision and well-being of the patient.

## Research methods and design

### Patients and methods

A retrospective review of patients who underwent parathyroid exploration in Mahatma Gandhi Medical College and Hospital between January 2023 and May 2024 was performed. This study was longitudinal and observational, obtaining information from the picture archiving and communications system (PACS) and clinical files. Retrospectively obtained demographic and clinical data were examined. Details of surgical records and pathology reports were also collected. A total of 44 patients underwent pre-operative 4DCT imaging. Patients who underwent 4DCT but did not undergo surgery were excluded from the study. The CT scans and surgical findings were evaluated to identify the role of 4DCT as a first-hand diagnostic tool in locating the abnormal parathyroid gland.

### 4DCT protocol

4DCT imaging was performed with a 128 slice CT machine (GE Optima CT660) as a multiphase study of the neck region. A pre-contrast scan was acquired, followed by the arterial phase, early and delayed venous phases either by bolus tracking or at 20 s, 60 s and 90 s, respectively, after the commencement of contrast injection. The fourth dimension is time (Table 1).

**TABLE 1:** Protocol of 4-dimensional computed tomography.

Position of patient	Placed supine with chin up to decrease the beam hardening artefact
Contrast used	Omnipaque 350 mg iodine/mL. Approximately 60 mL – 70 mL of contrast was used. For patients above 60 kg, 1 mL/kg of contrast was used. Contrast injected at 4 mL/s, followed by 25 mL of saline chase to eliminate any artifact due to contrast pooling in veins.
Scan area and field of view	From the maxilla to carina Approximately 25 cm
Contrast timing	Either through bolus tracking or automated time sequencing
Arterial and venous phase preceded by plain non-contrast scan	20 s and 60 s approximately after the start of injection
Thickness (mm)	0.625
Tube voltage (kVp)	120
Tube current (automatic exposure control) (mA)	Minimum 140, maximum 220
Software used to reduce radiation exposure	ASIR (adaptive statistical iterative reconstruction) and automatic exposure control system (Auto MAS) from GE Healthcare.

### Statistical analysis

Microsoft Excel 2019 (Microsoft, Redmond, Washington, United States) was used to gain insight into the data. The mean patient age, gender distribution, percentage of the single and multiglandular disease as well as laterality of the adenoma was calculated. The percentage of adenoma in eutopic and ectopic location was also calculated. True positive and sensitivity values were determined.

### Ethical considerations

The institutional ethics committee approved this study (reference no.: NO/MGMC&H/IEC/JPR/2024/4037) on 20 July 2024, and granted an exemption for the need for informed consent because of the retrospective nature of the study.

## Results

A total of 50 patients underwent 4DCT imaging at our radiology department between May 2022 and May 2024. Six of these patients did not undergo surgery because of comorbidities and asymptomatic patients who refused surgery. Thus, 44 patients were included in this study, of which 32 (72.7%) were females with a male: female ratio of 1:2.6. The mean calculated age was 50.3 ± 14.2 years.

Of the 44 patients, the 4DCT findings of three patients were discordant with the surgical findings, resulting in a calculated 4DCT sensitivity of 93%. In one of the patients, the lesion was missed because of an intra-thyroidal ectopic location and an atypical enhancement pattern. The thyroid gland itself was heterogeneous and appeared hypodense on non-contrast CT. In another patient, the 4DCT was negative, and bilateral neck exploration was performed, noting multiglandular disease. Similarly, in another patient, two abnormal parathyroid glands were reported on 4DCT, so bilateral neck exploration was performed, and three abnormal glands were identified, all of which were surgically removed.

Thirty-six of the 44 patients (81.8%) had a single parathyroid lesion, and 8 patients (18.1%) had multiglandular disease, which was consistent with the operative and histopathology results. Eight patients had multiglandular disease, which was not accurately diagnosed in two patients where bilateral neck exploration had to be performed with monitoring of the intraoperative fall of parathyroid hormone at 10 min and 20 min post-removal of the gland. The sensitivity of 4DCT in detecting multiglandular disease was 75%.

**FIGURE 2:**
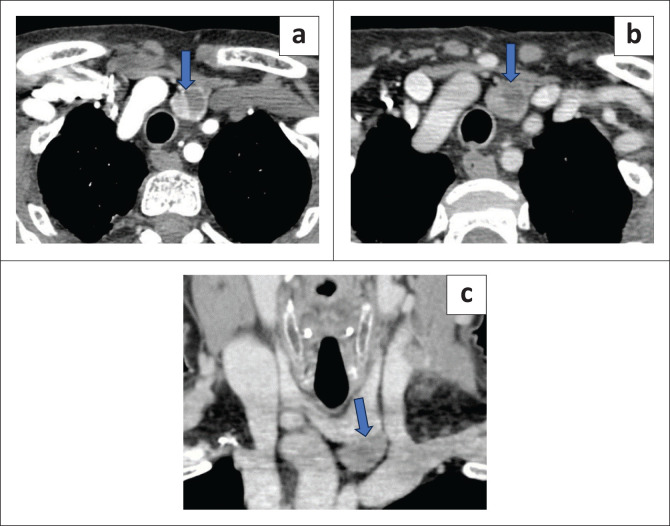
Heterogeneously enhancing mass lesion at the inferior aspect of the left lobe of the thyroid gland with an internal non-enhancing cystic component. Histopathology revealed a left inferior parathyroid adenoma. (a) Arterial phase axial scan demonstrated an enhancing lesion (blue arrow). (b) Venous phase axial scan showed partial washout (blue arrow). (c) Coronal delayed venous phase showed washout of the lesion (blue arrow).

Of the diagnosed parathyroid lesions, 52.1% were on the left, 35.4% on the right and 12.5% bilateral. Additionally, 76% were seen inferiorly and 24% superiorly. The most frequent site of a parathyroid lesion was the left inferior location (Figure 2).

**FIGURE 3:**
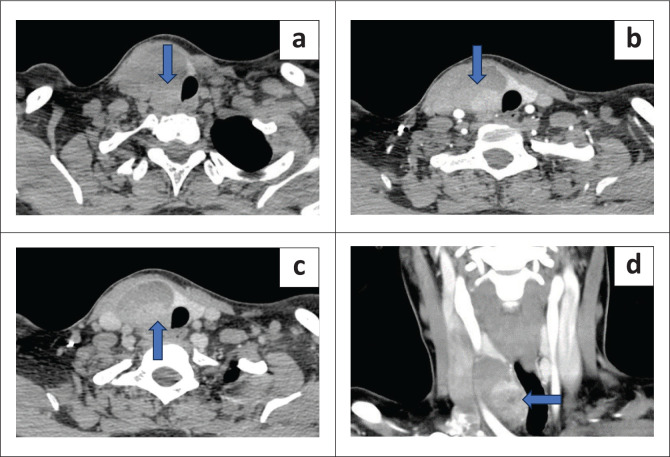
Heterogeneously enhancing mass lesion in the right lobe of the thyroid gland with a large hypodense component and an enhancing nodular component. Histopathology revealed an intra-thyroidal parathyroid adenoma. (a) Non contrast axial scan showed the lesion in the right lobe (blue arrow). (b) Arterial phase axial scan indicated an enhancing nodule in the lesion (blue arrow). (c) Delayed venous phase demonstrated washout in the nodule (blue arrow). (d) Coronal scan in the venous phase showed the enhancing nodule in the intra-thyroidal adenoma (blue arrow).

**FIGURE 4:**
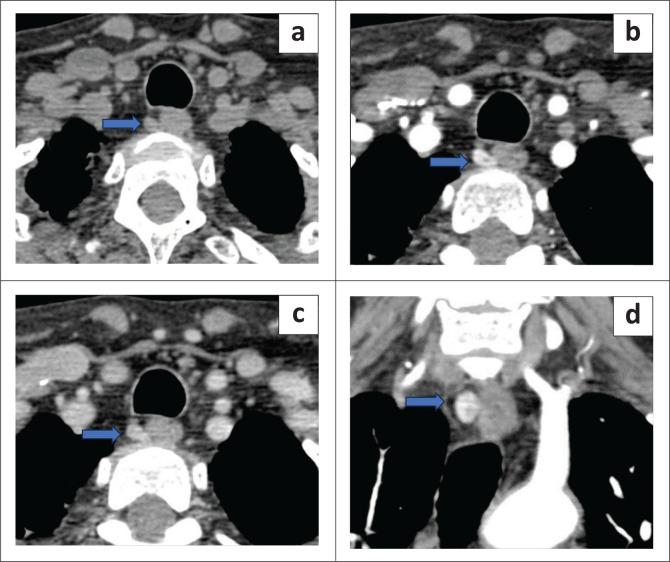
Ectopic parathyroid lesion in the right paraoesophageal region. (a) Non contrast axial scan showed the lesion (blue arrow). (b) Arterial phase axial scan shows enhancement (blue arrow). (c and d) Axial and coronal scans showing washout in the venous phase (blue arrows).

**FIGURE 5:**
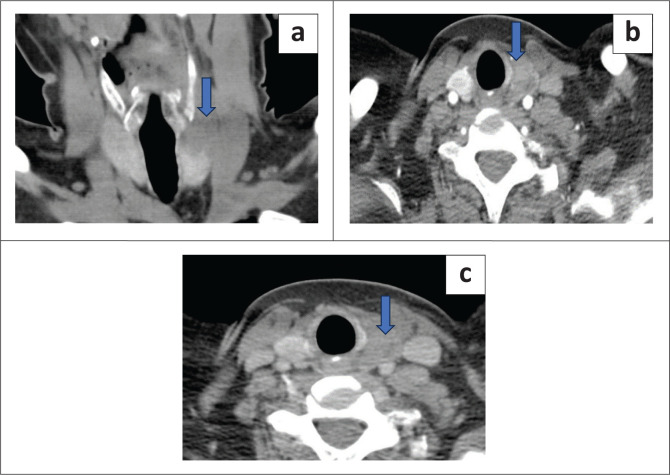
Heterogeneously enhancing mass lesion in the left lobe of the thyroid gland. Histopathology diagnosed an intra-thyroidal parathyroid carcinoma. (a) Non-contrast coronal scan demonstrated the lesion (blue arrow) appearing hypodense. (b) Arterial phase axial scan showed an enhancing lesion (blue arrow). (c) Axial delayed venous phase showed washout of the lesion.

Three of the 44 patients had parathyroid glands in ectopic locations. 4DCT located one ectopic lesion in the right thyroid gland (Figure 3) and another in the right paraoesophageal region (Figure 4). A second lesion in an intra-thyroidal location in the left lobe was missed on 4DCT, mistakenly reported as a suspicious thyroid lesion but histology revealed an intra-thyroidal parathyroid carcinoma (Figure 5).

**FIGURE 6:**
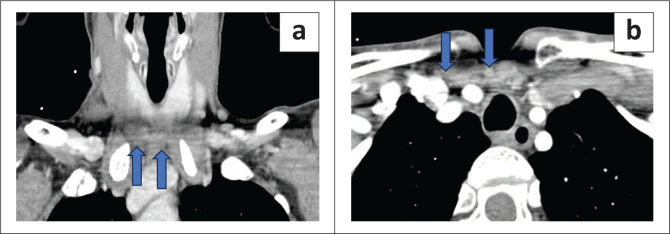
Few nodular lesions seen along the anteroinferior aspect of the thyroid gland marked by blue arrows in (a) and (b) suggestive of parathyromatosis. The patient had a history of previous parathyroid surgery.

One of the patients had previous surgery performed elsewhere without preoperative imaging and presented with increased serum calcium levels and suspected recurrence. Subsequent 4DCT at our institute revealed several small nodules at the inferior aspect of the thyroid gland, indicating parathyromatosis (Figure 6). Parathyromatosis is uncommon and results in recurrent hyperparathyroidism, marked by the development of multiple nodules of overactive parathyroid tissue in the neck and mediastinum. The USG and sestamibi (MIBI) scan for this patient, performed elsewhere, were inconclusive.

Aberrant origin of the right subclavian artery was seen in two patients. This anatomical variation suggests an altered course of the RLN, which is important for the operating surgeon to be aware of to avoid nerve injury.

4DCT also provided the extent and details of the surrounding anatomy. In one patient, a heterogenous thyroid gland with a suspicious supracricoid nodule was reported, diagnosed at histopathology as a papillary thyroid carcinoma with a metastatic nodule.

## Discussion

In preparation for parathyroidectomy, imaging is performed to assist with operative planning.^16^ It should be stressed again that primary hyperparathyroidism is not diagnosed on the basis of imaging.^2^ The primary imaging techniques utilised to evaluate primary hyperparathyroidism include ultrasonography (USG), sestamibi scan and 4DCT. Other imaging modalities are MRI and choline PET-CT.

When reporting the imaging findings, the role of the radiologist is to provide specific information about the total number of lesions, the lesion location and size, its depth relative to the skin surface, its relationship with nearby structures such as the thyroid gland, trachea, oesophagus, strap muscles and major vessels, and any anatomical variations, if present. The vertebral bodies are not considered important landmarks by the surgeon; hence the radiologist should refrain from using these. If the vascular supply to the lesion in the form of a polar vessel is visualised, then this should be included in the report.^17,18^ A polar vessel is observed in as many as two-thirds of these cases on 4DCT scan.^15^ The polar vessel, if identified, helps the radiologist in diagnosing parathyroid adenomas with more confidence.^5^

Ultrasonography is advantageous because of its easy accessibility, affordability and absence of ionising radiation. However, its main utility lies in the detection of sizable adenomas in their anticipated normal location. Ultrasonography has some disadvantages, as it is operator dependent and has limitations in identifying the adenomas in ectopic locations. The sensitivities for single-gland disease (SGD) fall within the range of 72% to 89%.^19^ Parathyroid hormone estimate combined with fine-needle aspiration (FNA) can be used for the diagnosis of parathyroid lesions.^19^ There is about a 5% risk of associated complications such as fibrosis and seeding with FNA. It is therefore only performed in cases with inconclusive findings.^20^

Conversely, the sestamibi scan is more sensitive in identifying individual adenomas and ectopic lesions, but it is less effective in identifying tiny lesions and multiglandular disease. In a meta-analysis, the pooled sensitivity of 99mTc-sestamibi single photon emission computed tomography (SPECT) for SGD (78.9%) was comparable to the pooled sensitivity of USG (76.1%).^21^ The sensitivity (81%) was significantly improved by combining 99mTc-sestamibi scintigraphy with USG.^22^ Compared to USG, 99mTcsestamibi scanning is also able to identify possible ectopic sites with greater sensitivity.^23^

MRI has lower spatial resolution than 4DCT and acquisition times are longer. PET-CT is another promising imaging technique, which has better spatial resolution than SPECT but is less available, expensive, and the tracer (fluorocholine/methionine) uptake is non-specific.

For the 4DCT scan, the pre-contrast scan in the protocol is important as it has been noted that without this phase, up to 25% of parathyroid lesions may go undetected.^24^ Parathyroid adenomas may exhibit lower enhancement than the thyroid during the arterial phase, or they may not display washout in the delayed phase.

A classic adenoma exhibits peak enhancement, which is about 138 Hounsfield units (HU) – 180 HU in the arterial phase on 4DCT images, which is higher than the thyroid tissue, and subsequent washout is seen in the delayed venous phase, resulting in reduced attenuation as compared to the thyroid gland.^2^ Sometimes, the peak enhancement of the parathyroid gland is seen in an early venous phase. It has been observed that approximately 20% of the parathyroid lesions demonstrate the classic enhancement pattern.^5^ Besides the varied enhancement patterns, adenomas can also show calcifications, cystic changes and sometimes fat deposition.

Important differentials to be kept in mind while searching for the parathyroid adenomas are lymph nodes, thyroid nodules and ectopic glands. Presence of exophytic thyroid tissue such as Zuckerkandl tubercle on post-contrast scan can be confusing and may lead to false-positive cases. On pre-contrast imaging, the tubercle can be characterised as it appears isoattenuating to the rest of the thyroid tissue due to the presence of iodine, thus limiting false-positive interpretation.^3^ The main differential of the adenomas are thyroid nodules which can appear hyperdense on the pre-contrast scan and lymph nodes that tend to demonstrate gradually increasing enhancement instead of the venous washout seen with parathyroid adenomas. Pre-contrast imaging is limited in cases of diffuse thyroid disease where the thyroid parenchyma no longer appears hyperdense.

The absence of a lesion on imaging in the setting of biochemically diagnosed hyperparathyroidism is also an indication of neck exploration surgery. Chances of multiglandular disease are increased when no obvious parathyroid lesions are seen on imaging as in one of the cases in this study. In multiglandular disease, often smaller parathyroid lesions are present. If the lesions are < 7 mm, then there should be a search for multiglandular disease. Those lesions measuring less than 7 mm showed 85% specificity for multiglandular disease.^25^ Similarly, lesions measuring greater than 13-mm size were found to have 85% specificity for single glandular disease.^25^ The sensitivity of detecting multiglandular disease in this study was 75%.

Parathyroid carcinoma is not very common and is difficult to diagnose preoperatively, but if the patient is of young age with very high levels of PTH and calcific foci are seen in the parathyroid, then parathyroid carcinoma can be suspected. The PTH levels are about 5 to 10 times higher or approximately > 500 mg/dL in carcinoma, whereas in adenomas, they may be within normal limits or mildly increased. Similarly, calcium levels are higher in cases of carcinoma (14.6 mg/dL – 15.0 mg/dL) as compared to adenomas (11.1 mg/dL).^26^

**FIGURE 7:**
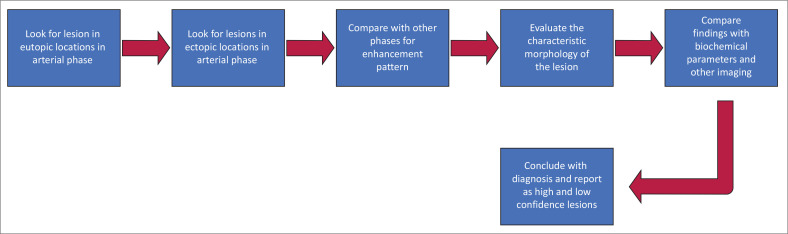
Flowchart depicting systematic approach to 4DCT.

The radiologist, while interpreting the images, may diagnose high and low confidence lesions in the same patient. If the intraoperative hormone levels do not decrease as expected after operating on the higher confidence lesion, it might be necessary to switch to a bilateral neck exploration. Hoang et al.^15^ summarised the approach for the detection of parathyroid adenomas as seen in the flowchart in Figure 7.

This study suggested that 4DCT can be a reliable investigation for the detection of parathyroid adenomas. The overall accuracy of 4DCT in identifying the location of adenomas was 93%.^27^ Rodgers et al. in their study found that 4DCT demonstrated higher sensitivity in determining laterality (88%) and quadrant of the neck (70%) compared to sestamibi SPECT (65% and 33%) and USG (57% and 29%), respectively.^14^

**FIGURE 8:**
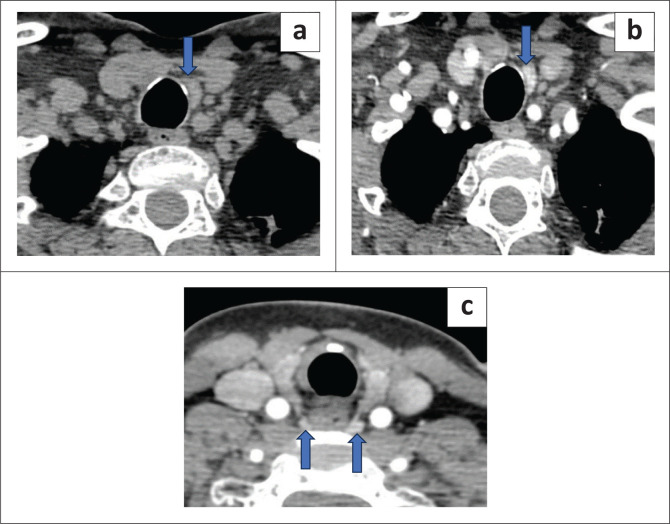
Multiglandular disease with a dominant left inferior parathyroid adenoma and hyperplasia of the other parathyroid glands. (a and b) Non-contrast and arterial phase axial scans demonstrate the left inferior parathyroid lesion (blue arrow). (c) Axial scan shows bilateral superior parathyroid lesions marked with blue arrows.

Specific research revealed that when analysing patients with multigland disease, 4DCT was found to be more effective than sonography and scintigraphy. The ability of 4DCT to detect multigland disease (32% – 53%) was still notably lower than its ability to detect SGD (88% – 93%).^14,25,28^ Multigland disease can be predicted with a high level of specificity when the lesions are small-sized, and serum biochemical markers also show low levels (Figure 8).^14^

Regional variability is clearly evident in the precision of imaging. When imaging is repeated after initially showing negative results in centres with high volume work, the sensitivity of detection of abnormal parathyroid gland can increase by up to 92%.^16^ Even though 4DCT scans offer these benefits, opinions on whether 4DCT should be used as the primary diagnostic method for locating parathyroid adenomas prior to surgery are divided. Some have recommended 4DCT as the main imaging technique for locating the diseased parathyroid gland before surgery.^29^ Few have suggested it to be used when other imaging is inconclusive.^30^ Some studies suggest that scintigraphy should be replaced by 4DCT,^28^ while some believe that scintigraphy plays a part in preoperatively locating the diseased gland.^31^ According to this model, using multiple imaging studies before a parathyroidectomy generally results in lower costs because it reduces the chance of bilateral exploration.^32^

A combination of USG and sestamibi scan can be used as primary investigation for the detection of parathyroid disease. 4DCT can be performed as complementary imaging to USG to look for an adenoma when the latter is inconclusive. 4DCT is also preferred in cases of clinical or USG doubt. Despite the additional expenses in the diagnostic process, multiple research studies have shown that patients undergoing 4DCT experience reduced surgical durations, leading to the overall cost-effectiveness of this imaging technique.^33,34^

4DCT was considered as a substitute for traditional imaging methods, but institutions with successful scintigraphy results may not find it necessary to incorporate 4DCT into their protocol. However, once radiologists become accustomed to 4DCT scans, there is potential for it to be integrated into the institutional protocol. If it becomes the favoured imaging method, referring physicians and radiologists may begin to utilise it more frequently as the preferred imaging modality. In a 2015 survey, radiologists who participated and had more than 3 years of experience were more inclined to use 4DCT as their primary imaging modality.^34^ The percentage of patients undergoing 4DCT scans at one such high-volume institution rose from 1.5% to 75.8% from 2009 to 2014.^27^

### Radiation exposure and relative contraindications

The radiation dose of 99mTc-sestamibi scanning is less than that of 4DCT imaging. In the research conducted by Mahajan et al.,^35^ the effective radiation dose for 4DCT and parathyroid scintigraphy was 10.4 mSv and 7.8 mSv, respectively. However, the risk of cancer over a lifetime was found to be minimal in the age group of patients who usually develop primary hyperparathyroidism. Hoang JK et al.,^36^ also noted that the impact on lifetime cancer risk is minimal compared to baseline cancer rates. Clinicians should therefore make decisions about workup in this age group of patients without allowing concerns about radiation-induced risks. Typically, the average patient diagnosed with hyperparathyroidism is a woman over the age of 50 years. Within this demographic, the lifetime risk of developing thyroid cancer related to 4DCT imaging is 4 cases/100 000. This is about three times less than the incidence of thyroid cancer in the 2009 population documented at 14/100 000.^15^ This, however, does not hold true if the individual undergoing 4DCT is aged around 30 years or less. In this study, four patients were aged 30 years or less.

When evaluating the lifetime risk associated with this procedure, the benefits of minimally invasive focused surgery compared to bilateral neck exploration and the identification of a symptomatic parathyroid adenoma by 4DCT surpasses the radiation risk in the younger patient group. It is crucial to remember that the majority of these patients will never need another parathyroid 4DCT scan in their lifetime because surgery is usually curative.^3^ The total number of phases acquired in 4DCT scan can be reduced to decrease the radiation dose. Calculation of the percentage arterial enhancement using non-contrast and arterial phase scans may be helpful in the diagnosis.^37^

The authors are considering creating a simplified protocol to reduce the number of CT phases. Rather than the standard non-contrast, arterial, early and delayed venous phases, the plan is to determine the percentage arterial enhancement, utilising the HU value in the arterial phase. This allows for the development of a simple two-phase protocol, thereby exposing patients to less radiation. Consequently, the delayed phase may also be omitted. Current studies implementing these revised protocols in newer patients are being conducted to optimise imaging and reduce radiation exposure during 4DCT.

The limitations of this study are small number of patients in the sample and selection bias as all patients and 4DCT scans have been performed in the same institution.

## Conclusion

4DCT imaging is useful for the detection of parathyroid lesions, especially in cases of solitary adenomas and reoperative cases when there is distorted neck anatomy from prior surgery. It is also useful in cases with co-existent disease of the thyroid gland and adjacent structures. No definite guidelines are available presently with recommendations for the use of any single or combination of imaging modalities. Radiological work-up depends on the institutional setup and individual preferences. With time and experience, results with 4DCT are likely to improve.
